# The Work of the Imperial Cancer Research Fund

**Published:** 1905-09-16

**Authors:** 


### HOSPITAL CLINICS.

### THE WORK OF THE IMPERIAL CANCER RESEARCH FUND.

(*Continued from p.* 412.)

As a result of a careful comparison of the features of propagated cancer, and the sporadic disease, the authors of the report[1] under notice are able to formulate the important conclusion that propagated cancer reproduces all the characteristic features of the sporadic and naturally-occurring disease. Although transplantation throws at present no light upon the processes by which cells acquire the property of malignancy, it assists in the separation of essential from incidental features of their growth when malignant. The enormous total mass of growth which may be attained by successful inoculation through several generations of mice shows that the limits of growth of sporadic tumours are not set by the bulk which suffices to destroy an individual animal. It is stated in the introduction to this volume that a carcinoma has been propagated in mice for three and a half years since the death of the animal supplying the original material —that is to say, for a period exceeding the duration of life in a mouse. Enough tissue has been thus produced to have sufficed for the building up of 1,500 adult mice had the cells developed into the organs of individuals. Had they, on the other hand, developed into one animal, that mouse would have been as big as a St. Bernard dog. This proliferative power is inherent in all malignant growths, whether in animals or man. Examination of early cancers, of which some excellent reproductions are given to illustrate the text, shows no evidence of the conversion of healthy tissues into malignant ones, but rather of a minute local establishment of malignancy, with subsequent intercalation of proliferated tumour-cells among remnants of normal structure. Thus again the essential feature of malignant extension is the proliferation of the parenchyma.

The experiments of the fund have demonstrated a wide variation in the behaviour of propagated tumours of similar origin. Thus the degree of necrosis and the rate of growth observed in the descendants of the same tumour-stock, are susceptible of great differences in different individual mice. In addition to these variations in contemporaneous tumours of the same stock, secular variations occur. Thus, in the early propagations from Professor Jensen's tumour made in London, necrotic changes were extremely rare. This state of affairs was followed by a long interval, during which extreme necrosis was almost a constant feature of the growths produced, while more recently again a reversion towards the early features has become apparent. The existence of such secular variations is a discovery of high importance, for it is easy to see how much they may bear upon the interpretation of reputed cures by means of cytolytic sera and otherwise. In addition to these secular variations in the histological characters of the tumours, the results of long-continued experiments show gradual fluctuations in the rate of growth, and the percentage of successful inoculations; fluctuations which seem sufficiently ordered to justify the conclusion that the power possessed by malignant cells of establishing themselves in new hosts, varies periodically from inherent causes. If, as the present experiments indicate, this periodical variation is the expression of a fixed law, the fact may explain the dependence of the growth of sporadic and propagated tumours on time limitations in different animals. It may be that we have to do with a naturally cyclical form of growth.

Much has been said and written about the influence of radium upon malignant new-growths. This influence, which is undoubted, has been generally credited to a selective action of radium upon the tumour-cells. In order to set the matter at rest if possible, the Fund has carried out a number of experiments. It was found that both radium applied in a solid state, and also sterilised distilled water rendered radio-active and injected into the tumour, could produce a diminution in the size of the latter. Examination of the histological changes showed that extravasation of blood was an early and constant feature. It appears within the first four days, and is followed by an inflammatory reaction in the connective tissues surrounding the growth, the tumour-cells being at this time entirely unaffected. Later the tumour-cells become broken into small groups by strands of young fibrous tissue, and the cells themselves become vacuolated. This vacuolation is probably due to cicatricial interference with the blood-supply. Control experiments were carried out with large doses of adrenalin, in the hope that the general rise of blood-pressure might result in hæmorrhage from the thin-walled capillaries of the new growth which are unprovided with a vasomotor apparatus. In one animal so treated the tumour did undergo a diminution in size, and presented to the microscope appearances identical with those seen at a similar stage of the processes following the injection of radio-active fluids. The conclusion is that radium has no selective action upon tumour-cells, for in the first place results similar to those obtained with radium have been obtained with adrenalin; secondly, tumours which have undergone prolonged exposure to radium have, when inoculated, produced daughter-tumours of ordinary character; again, in the rare case of spontaneous arrest of growth in Jensen's tumour, the histological characters of the change are identical with those observed in the case of radium and adrenalin treatment, namely a primary inflammatory reaction in the supporting structures of the tumour. The truth is probably that in every case the first element of alteration is a local extravasation of blood, which calls forth in the neighbourhood of the tumour-cells an inflammatory reaction leading to new connective tissue formation, and ultimate strangling of the growth by cicatricial contractions.

(*To be concluded.*)

[1] Scientific reports of the Imperial Cancer Research Fund. Part II. London. 1905. Price 2s. 6d.

